# Comprehensive Meta-Analysis of COVID-19 Global Metabolomics Datasets

**DOI:** 10.3390/metabo11010044

**Published:** 2021-01-09

**Authors:** Zhiqiang Pang, Guangyan Zhou, Jasmine Chong, Jianguo Xia

**Affiliations:** 1 Institute of Parasitology, McGill University, 21111 Lakeshore Road, Ste Anne de Bellevue, QC H9X 3V9, Canada; zhiqiang.pang@mail.mcgill.ca (Z.P.); guangyan.zhou@mail.mcgill.ca (G.Z.); jasmine.chong@mail.mcgill.ca (J.C.); 2 Department of Animal Science, McGill University, 21111 Lakeshore Road, Ste Anne de Bellevue, QC H9X 3V9, Canada

**Keywords:** COVID-19, metabolomics, mass spectrometry, meta-analysis, coronavirus

## Abstract

The novel coronavirus SARS-CoV-2 has spread across the world since 2019, causing a global pandemic. The pathogenesis of the viral infection and the associated clinical presentations depend primarily on host factors such as age and immunity, rather than the viral load or its genetic variations. A growing number of omics studies have been conducted to characterize the host immune and metabolic responses underlying the disease progression. Meta-analyses of these datasets have great potential to identify robust molecular signatures to inform clinical care and to facilitate therapeutics development. In this study, we performed a comprehensive meta-analysis of publicly available global metabolomics datasets obtained from three countries (United States, China and Brazil). To overcome high heterogeneity inherent in these datasets, we have (a) implemented a computational pipeline to perform consistent raw spectra processing; (b) conducted meta-analyses at pathway levels instead of individual feature levels; and (c) performed visual data mining on consistent patterns of change between disease severities for individual studies. Our analyses have yielded several key metabolic signatures characterizing disease progression and clinical outcomes. Their biological interpretations were discussed within the context of the current literature. To the best of our knowledge, this is the first comprehensive meta-analysis of global metabolomics datasets of COVID-19.

## 1. Introduction

COVID-19 is an unprecedented health emergency driven by the severe acute respiratory syndrome coronavirus 2 (SARS-CoV-2) [1]. This disease had led to over 1.2 million deaths globally by 5 November 2020 according to the WHO [2]. A broad spectrum of clinical presentations has been observed, ranging from asymptomatic, mild, moderate, or severe symptoms, to fatal illness. Such diverse trajectories are believed to be the result of the differences in individual immune responses to COVID-19 [1,3,4]. A comprehensive understanding of the molecular events underlying different clinical courses is urgently needed to help improve patient management and to accelerate the development of therapeutic strategies.

Metabolism fuels all biological processes in the human body, including immune responses. Blood metabolites are the end products of many systematic processes and are informative indictors of biochemical activities or diseases’ phenotypes [5,6]. Powered by the growing applications of high-resolution mass spectrometry (MS), metabolomics has become a key member of the omics toolkit in biomedical research. Multiple metabolomics studies have been recently conducted across the world to study COVID-19, revealing key metabolic dysregulations during the disease’s progression [7,8,9,10,11,12,13,14,15,16]. For instance, several amino acids have been observed to be positively correlated with the severity of COVID-19 as key indicators of clinical prognosis of the disease [8,9,12,13,14]. Perturbations in energy metabolisms such as glycolysis and pentose phosphate pathway, TCA and urea cycle have also been reported [7,9,12]. The changes in lipid metabolites such as fatty acid, arachidonic acid, glycerophospholipid and sphingolipids are now considered important hallmarks in the pathogenesis of COVID-19 [17,18]. To help to accelerate diagnostics, prognostics, and treatment of the disease, the COVID-19 MS Coalition has been recently launched as a collective community effort to combat the pandemic [19].

Meta-analysis of the available datasets is a promising approach to gain a comprehensive understanding of the pathogenesis of the disease [20,21], as well as to help to identify robust biomarkers to inform better clinical care and to facilitate therapeutics development. Indeed, meta-analyses of the COVID-19 transcriptomics datasets are quickly emerging and have produced important insights into common and unique gene expression patterns of the disease [22,23,24]. However, to the best of our knowledge, meta-analyses of COVID-19 metabolomics datasets have not been conducted so far. This could be due to a much smaller number of metabolomics studies reported so far or even more likely, due to the practical challenges in dealing with the high levels of heterogeneity inherent in global metabolomics datasets. Unlike transcriptomics in which genes or transcripts can be reliably identified and quantified directly from sequencing data, the features reported by liquid chromatography (LC)-MS-based global metabolomics are peaks characterized by their retention times and m/z values, which are insufficient for metabolite identification in general. Moreover, spectral peaks are not usually comparable across different studies due to differences in chromatographic and/or MS conditions.

To address this research gap and to gain a better understanding of the metabolic changes underlying the disease, we systematically collected the COVID-19 global metabolomics datasets that were publicly available as of 5 November 2020 and implemented a computational pipeline for spectra processing, visual exploration and meta-analysis. In this manuscript, we report our findings and discuss their implications within the context of the current understanding of the disease.

## 2. Results

### 2.1. Summary of Different Datasets and Their Clinical Characteristics

**Figure 1:**
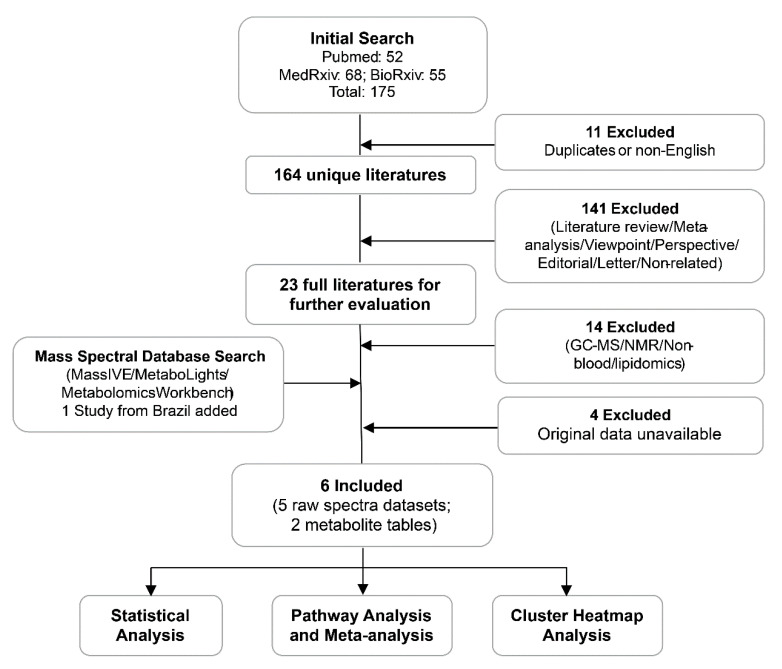
The workflow diagram of our data curation process and analysis strategy. The six studies contain seven datasets, five as raw spectra and two as putatively annotated peak tables.

A total of 175 COVID-19 papers were identified in our initial search. After filtering these studies based on our inclusion/exclusion criteria, six studies from the USA, China and Brazil were finally included in this meta-analysis (Figure 1). One study from the USA generated two datasets using two different metabolomic platforms. As a result, seven datasets were finally included in this meta-analysis. Among them, five datasets were obtained as raw spectra, including two from MetaboLights [25], one from MassIVE (https://massive.ucsd.edu/) and two directly from the authors. The remaining two datasets were annotated metabolite intensity tables obtained from the Supplementary Materials of the original publications. In total, 438 samples from 337 subjects were included. Table 1 summarizes the key information about these datasets. More details on the patient classification criteria, technical information on experimental conditions and the demographic characteristics of all subjects are provided in Tables S1–S3, respectively.

### 2.2. Processing and Overview of Individual Datasets

**Figure 2:**
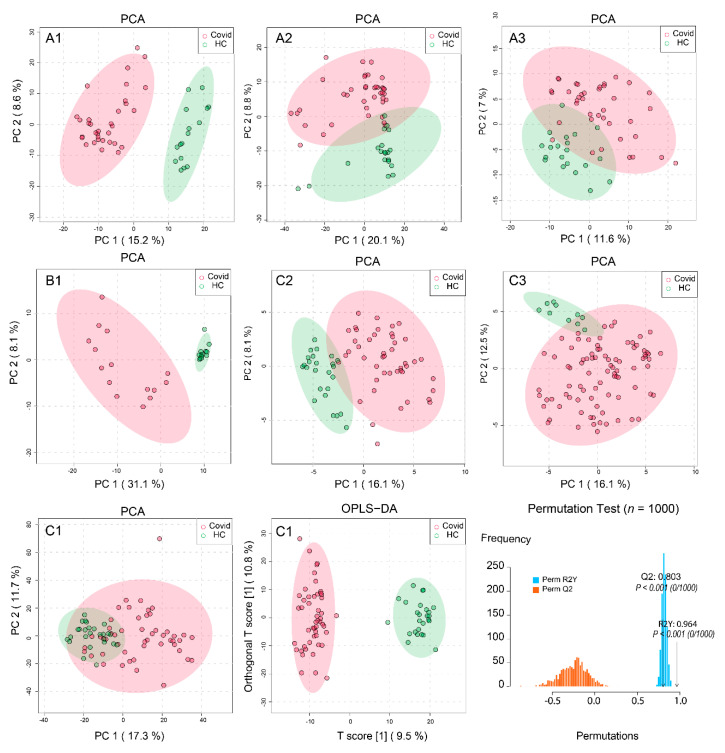
Overview of the separation patterns between COVID-19 and healthy controls (HCs) across the seven datasets. The 1st and 2nd rows are the principal component analysis (PCA) results of datasets **A1**, **A2**, **A3**, **B1**, **C2** and **C3**, respectively. For **C1** (3rd row), we performed PCA, followed by Orthogonal Projections to Latent Structures Discriminant Analysis (OPLS-DA) and its validation by permutations (*n* = 1000).

The five raw spectra datasets were processed using our MetaboAnalystR 3.0 pipeline for optimized peak detection, quantification and alignment (with peak numbers ranging from 2553 to 11,665). The final optimized parameters are provided in Table S4. The resulting peak intensity tables from both positive and negative ion modes were combined, median normalized and log transformed for an initial data quality check and visual inspection. The two annotated peak tables were directly used to perform multivariate analysis. Figure 2 shows the results from Principal Component Analysis (PCA) of samples between COVID-19 and healthy controls (HCs). No clear batch effects were observed in the normalized datasets. The first two PCs showed the clear patterns of separation for all datasets (except the C1). The relative low variances explained by the top two PCs could be due to the very high dimensionality of the global metabolomics data, similar to PCA of transcriptomics data. We further analyzed C1 using Orthogonal Projections to Latent Structures Discriminant Analysis (OPLS-DA), which showed a significant separation. The model was evaluated with cross validations (Q2 0.964 and R2 0.803) and permutation tests (*p*-value < 0.001). Overall, these results indicated overall significant metabolic perturbations in COVID-19 patients across all study populations. For two randomly selected datasets, we also compared the results from our spectra processing pipeline against those from two other public spectra processing tools [27,28] and observed that the PCA from our pipeline produced better separation patterns (data not shown).

### 2.3. Metabolic Pathways Changes in COVID-19 Patients

**Figure 3:**
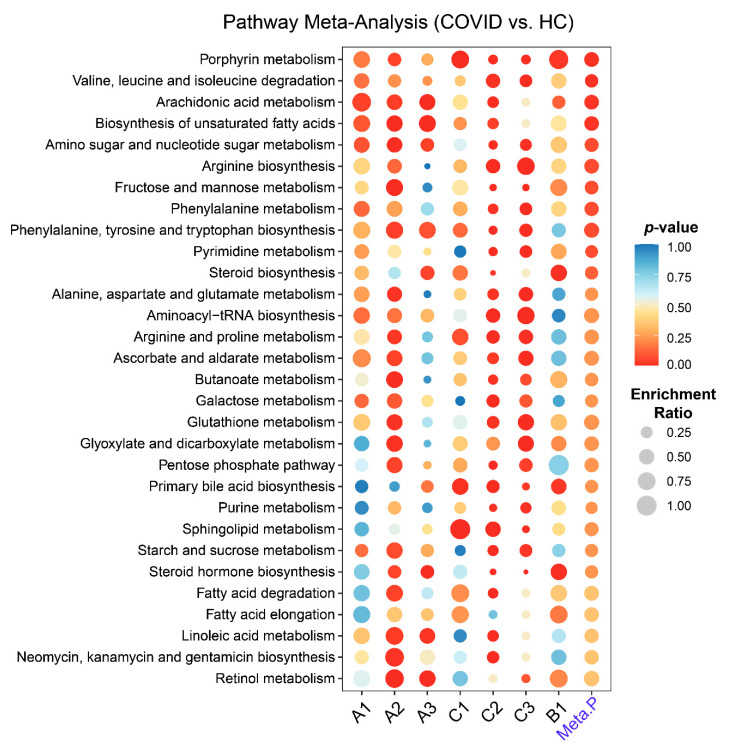
Pathway analysis and meta-analysis between COVID-19 and healthy controls (HC) across the seven datasets. Each row represents a pathway and each column represents a dataset. The rightmost column shows the result from the meta-analysis.

For each raw spectral dataset, we performed metabolic pathway activity predictions using the Mummichog approach [29] in MetaboAnalystR 3.0. For the two annotated peak tables, we performed pathway analysis using the quantitative enrichment method based on their annotations. The human Kyoto Encyclopedia of Genes and Genomes (KEGG) pathway database was used in both cases. The pathway-level *p*-values were further integrated to produce a final ranked list of perturbed pathways (Figure 3). Four common pathways were significantly changed between COVID-19 patients and HCs (*p*-value < 0.05). Despite the ambiguities in individual compound assignments, we also attempted to extract the peaks underlying these four perturbed pathways from individual studies. The correlations between these peaks with the symptom onset days were then statistically evaluated. Nine peaks were significant (*p*-value < 0.05), with one negative and eight positive associations (Figure S1).

### 2.4. Identification of Metabolic Hot Spots in COVID-19

**Figure 4:**
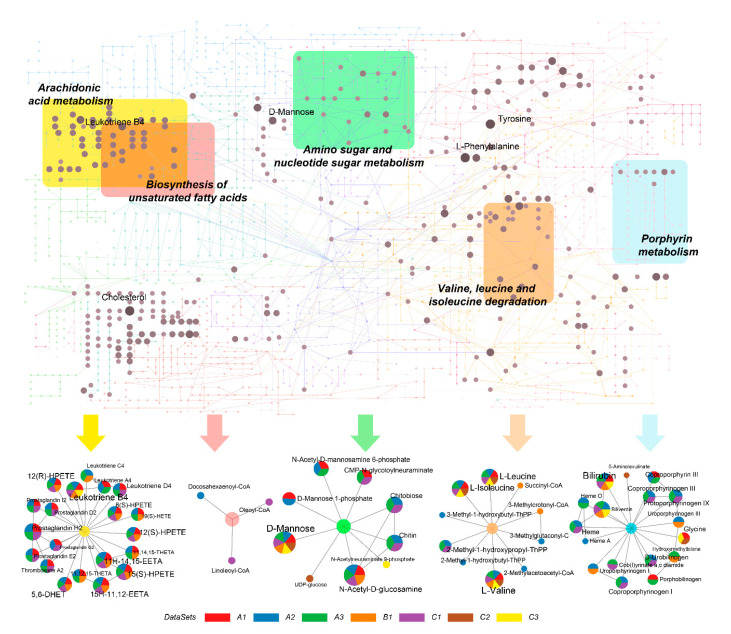
Overview of potentially perturbed metabolites and extracted metabolic pathways based on the seven datasets. The top five pathways ranked by their integrated *p*-values are shown here. The top part is the KEGG global metabolic map, with nodes in brown showing the matched metabolites whose sizes are based on the total number of hits from different datasets. Different colored areas represent different pathways. At the bottom are the five extracted pathways corresponding to the five colored regions in the map, with nodes shown as pie charts whose sizes and components correspond to the hits from different datasets.

In order to gain a high-level overview of the changes in metabolic activities caused by COVID-19, we mapped all significant metabolites (based on putative peak annotations) onto the KEGG global metabolic network (Figure 4). Network visualizations could reveal coordinated metabolic activities as clusters of metabolites distributed both within and across pathway boundaries. A total of 65 compounds have been reported by at least two datasets within these pathways. The five colored areas indicate the top five pathways identified in Figure 3. Other metabolic pathways also contain many metabolites that have received hits from multiple datasets. For instance, cholesterol, d-Mannose, Tyrosine, L-phenylalanine and Bilirubin are the top five most common compounds identified in our meta-analysis, which indicates their potentials as metabolic biomarkers. To complement the meta-analysis, we performed cluster heatmap analysis at feature levels for each dataset. We were able to identify clusters with consistently upregulated or downregulated metabolic patterns between the two conditions in six out of the seven datasets (Figures S2–S7). The pathway analyses based on these patterns reported similar results to those in Figure 3.

### 2.5. Metabolic Changes between Mild-to-Moderate (MM) and Severe COVID-19

**Figure 5:**
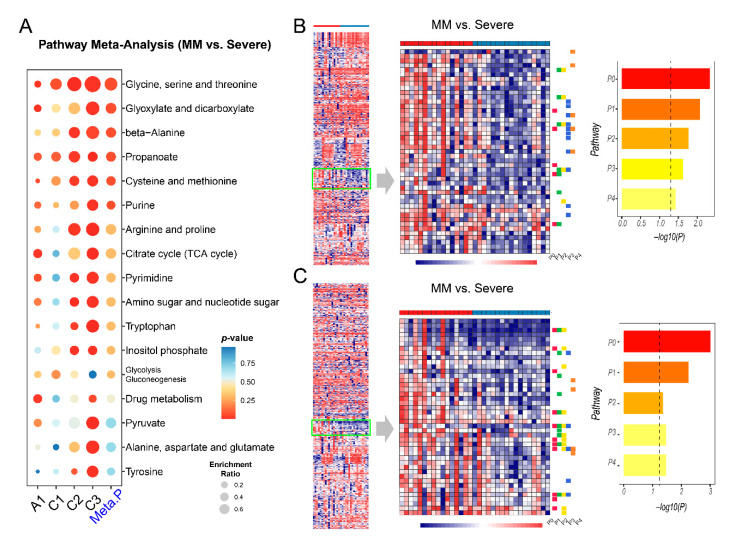
Metabolic pathway analysis and cluster heatmap analysis between mild-to-moderate (MM) and severe groups. (**A**) Summary of pathway analysis and meta-analysis result. (**B**) Enrichment analysis on a pattern of interest identified in dataset A1 (negative ion mode). P0: Caffeine metabolism; P1: Glyoxylate and dicarboxylate metabolism; P2: Citrate cycle (TCA cycle); P3: Purine metabolism; P4: Lysine degradation. The vertical dashed line in the bar plot is the threshold of *p* = 0.05. (**C**) Enrichment analysis on a pattern of interest in A1 (positive ion mode). P0: Glycine, serine and threonine metabolism; P1: Glyoxylate and dicarboxylate metabolism; P2: Cysteine and methionine metabolism; P3: Citrate cycle (TCA cycle); P4: Selenocompound metabolism.

Four datasets contain samples from patients classified as MM and severe COVID-19. The patients with fatal outcomes were also included in the severe group for this comparison based on their clinical status. We first aimed to identify commonly perturbed metabolic pathways across the four datasets. As summarized in Figure 5A, six pathways were ranked as the top changed metabolic pathways between MM and severe groups. Similarly, we also mapped the significant metabolites onto the KEGG global metabolic map and noticed that only a few metabolites (L-Alanine, Uridine and Uracil) were shared across the four datasets (Figure S8). We then performed cluster heatmap analysis on individual datasets and visually examined the cluster patterns to identify consistent changes between MM and severe COVID-19. As shown in Figure 5B,C, there are some regions that show a general decrease in abundance in the Severe group of A1. A total of eight metabolic pathways were significantly downregulated in this group. Similarly, a consistent metabolic pattern was also found in dataset C3 (Figure S9), but not in C1 and C2 (Figure S10).

### 2.6. Exploration of Metabolic Perturbations in Fatal COVID-19

**Figure 6:**
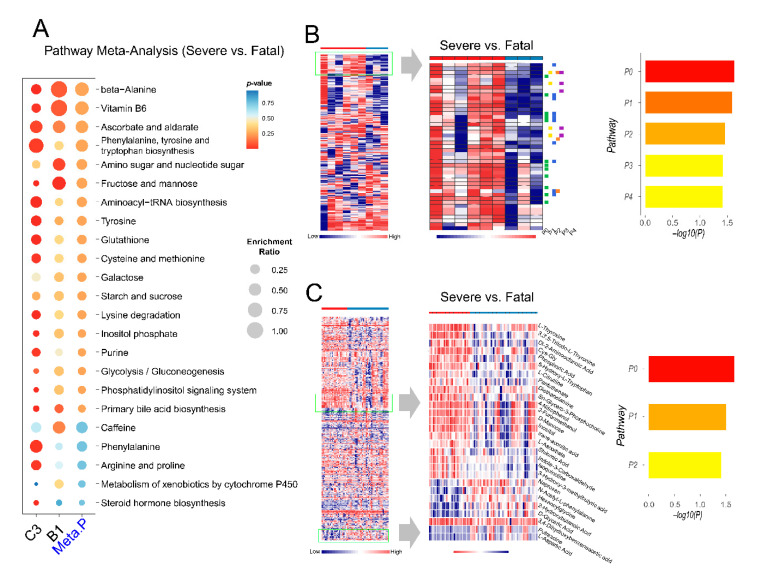
Pathway analysis and cluster heatmap analysis between severe and fatal groups. (**A**) Summary of pathway analysis and meta-analysis result. (**B**) Enrichment analysis on a metabolic pattern of interest in dataset B1 (positive ion mode). P0: Primary bile acid biosynthesis; P1: D-Glutamine and D-glutamate metabolism; P2: Steroid biosynthesis; P3: Ubiquinone and other terpenoid-quinone biosynthesis; P4: Alanine, aspartate and glutamate metabolism. The vertical dashed line in the bar plot is the threshold of *p* = 0.05. (**C**) Enrichment analysis on the combined metabolic patterns of interest in dataset C3. P0: Arginine biosynthesis; P1: Tyrosine metabolism; and P2: Pantothenate and Coenzyme A (CoA) biosynthesis.

Three datasets (C1, C3 and B1) contained COVID-19 patients with mortality information. The C1 dataset was excluded because it contained only two cases to perform meaningful statistical analysis. Metabolic differences between the severe and fatal patients were evaluated with the remaining two datasets (Figure 6). Several common metabolic pathway changes were identified from these two datasets (Figure 6A). Six metabolites were found as the common hits after mapping to the KEGG global metabolic map (Figure S11). From the cluster heatmap of the B1 dataset (positive ion mode), we identified a consistent pattern of change showing five enriched metabolic pathways (Figure 6B). From the cluster heatmap of C3, we combined two regions of interest and identified three enriched metabolic pathways (Figure 6C).

## 3. Discussion

Patients with SARS-CoV-2 infection manifest a classical respiratory virus-like clinical course with activated innate and adaptive immune responses [3,30]. Multiple metabolic pathways, such as amino acid metabolism, energy metabolism and lipid metabolism, are involved in the initiation and maintenance of the immune responses in COVID-19. Our meta-analysis has not only confirmed the dysregulations of these pathways as reported by original studies, but also observed novel patterns of metabolic changes underlying the pathogenesis of COVID-19.

Several common metabolic pathways were identified by comparing COVID-19 patients with healthy subjects. The most significantly perturbed pathway is Porphyrin metabolism or Heme biosynthesis, which is consistent with previous reports [31,32]. The SARS-CoV-2 virus could capture hemoglobin, displace iron and decrease the ability of carrying oxygen, thus causing respiratory distress and coagulation reactions, damaging multi-organs [33]. The hijacking of the cellular amino acid metabolism to fuel viral proliferation might be a critical mechanism underlying the COVID-19 pathogenesis [34]. Arachidonic acid is an endogenous bioactive antiviral lipid, and this metabolic pathway has been suggested to play an important role in susceptibility to COVID-19 [35,36]. The elevated levels of free poly-unsaturated fatty acids are characteristics of COVID-19 patients [10,37]. However, their roles are still controversial [38,39,40] and warrant further studies.

The heterogeneity of COVID-19 patients shows a wide spectrum of symptoms as well as disease severity. The risk categorization of COVID-19 is difficult because of the complexity of the pathophysiological status of the patients. Therefore, understanding the molecular underpinnings of the disease severities is important to help to reduce the mortality.

Patients with mild-to-moderate (MM) cases of COVID-19 typically have an optimistic prognosis and can recover very quickly. Pathway analysis between MM and severe COVID-19 showed six common perturbed pathways. Most of them were amino acids pathways. Our analysis identified propanoate as a novel pathway in the progression of COVID-19. Propanoate metabolism usually starts with the gut microbiota and enters into immune cells such as macrophages, thereby modulating the biological process [41]. The glyoxylate and dicarboxylate metabolism pathway has been reported to be decreased after infection [42]. We observed that this pathway was downregulated in severe compared to MM. Downregulation of the TCA might be related to the high energy consumption of SARS-CoV-2 [7]. Decreased TCA metabolism would cause an imbalance of anti-oxidization and inflammatory damage [43,44]. Finally, selenocompound is an ex vivo compound originating mainly from gut microbiota [45], and the biological effect of its decrease needs further investigation. Both propanoate metabolism and selenocompound metabolism suggest potential roles played by gut microbiota in the progression of COVID-19, a topic which has gained increasing attention recently [41,46].

SARS-CoV-2 infection can not only cause pathogenic changes in the respiratory system but can also lead to systematic multi-organ damages and death [47]. Preventing fatal COVID-19 is the most important objective in current clinical care. In addition to the observation of extensive dysregulations in amino acid metabolism, our analysis also detected other energy-related pathways such as mannose metabolism as reported previously [9]. The change in glutathione metabolism was observed in fatal COVID-19, providing direct evidence for a recent clinical hypothesis that glutathione deficiency could lead to serious manifestation and death in COVID-19 [48]. This metabolic pattern also reveals other interesting metabolic signatures. For instance, biosynthesis of bile acid might be a key clinical manifestation of liver damage by SARS-CoV-2 infection [7,49]. The inhibition of its synthesis might accelerate the deterioration of COVID-19 to death [50]. Endogenous steroid biosynthesis was found to be decreased, although it could have been caused by medical treatments. Ubiquinone has been reported to alleviate the cytokine storm and restore exhausted T cells in COVID-19 [51]. The suppression of its biosynthesis could worsen the disease condition. The role of vitamin B5 biosynthesis on the deterioration of COVID-19 remains unclear, but vitamin B6 has been proposed to ameliorate the severity of COVID-19 [52].

The high level of heterogeneity inherent in global metabolomics datasets poses tremendous challenges to conduct metabolomics meta-analysis at the feature (MS peaks) level. In this study, we utilized the well-established Mummichog method to first compute pathway activities from MS peaks and performed meta-analysis at the pathway level. There are however, several limitations to this analysis method. The potential bias caused by differences in the extraction procedures and analytical platforms at pathway level remains an open question. Due to the nature of putative annotations, the significant metabolites reported in this study need to be further validated using more targeted approaches. Although the potential confounding factors (diet, ethnicity, medical treatment, etc.) were controlled within each study, they were not considered in the current meta-analysis because most meta-data are incomplete or missing from the original studies. We intend to address this issue by expanding this analysis to include multiple-cohorts-based metabolomics studies when more datasets become available in the coming year. In addition, many signatures are likely to reflect general immune and inflammatory responses. We plan to include studies on other viral infections (such as SARS-CoV and influenza) to identify unique metabolic signatures of this disease as illustrated in a recent meta-analysis based on transcriptomics [53].

## 4. Methods and Materials

### 4.1. Data Curation

This meta-analysis was strictly conducted based on the PRISMA guidelines [54]. All studies were searched for on PubMed, medRxiv (www.medrxiv.org/), and bioRxiv (www.biorxiv.org/) using the search term “(COVID-19) AND (Metabolomics)” before 5 November 2020. The inclusion criteria for further processing were as below: (1) The study should have had a matched healthy control for COVID-19 samples; (2) all raw spectra data or original/annotated peak tables should have been available publicly or upon request; (3) to ensure comparability, only LC-MS-based global metabolomics datasets were included; other metabolomics datasets generated by gas chromatography (GC)-MS or nuclear magnetic resonance (NMR) were excluded. The PRISMA 2009 Flow Diagram is provided in Figure S12.

### 4.2. Patient Classification

All COVID-19 patients were diagnosed separately at their original hospitals or testing centers. Their disease severities were classified according to a combined standard based on the *Guideline of Diagnosis and Treatment Protocol for Novel Coronavirus Pneumonia (8th)* published by the National Health Commission of China [55], *WHO R&D Blueprint novel Coronavirus COVID-19 Therapeutic Trial Synopsis* [56], and an inflammation correlated cytokine, IL-6 as used in the original studies [12].

### 4.3. Raw Spectra Processing

Raw LC–MS spectra were first converted and centroided from vendor format to mzML using ProteoWizard [57]. All centroided spectra were processed with an automated pipeline with built-in parameter optimization procedures as described in MetaboAnalystR 3.0 [58]. For annotated peak tables, the names were standardized with the ID conversion tool in MetaboAnalyst [59]. The remaining ambiguous compounds/peaks were manually corrected based on HMDB [60].

### 4.4. Statistical Analysis

Chemometrics analysis (PCA and OLS-DA) was performed based on the normalized peak tables using the corresponding functions in MetaboAnalystR 3.0. Spearman correlations between the onset days of symptoms and metabolic features were calculated using base R package (v4.0.2). The confidence interval of the significant correlation was set to 0.95.

### 4.5. Metabolic Pathway Analysis and Meta-Analysis

The pathways analysis on the datasets from raw spectra in this present study was performed independently for every dataset using Mummichog [29] from the MetaboAnalystR 3.0 workflow [58]. The pathways analysis on the two annotated peak tables was completed with the Pathway Analysis module based on the default quantitative enrichment analysis method and the human KEGG database [61]. The meta-analysis was performed at the pathway levels. The combined *p*-values were computed based on the vote counting method in the metap package in R (v4.0.2) by counting the *p*-value from two directions and outputting an integrated *p*-value based on the counting results. The enrichment ratio describes the relative percentage of the empirical compound hits to the whole empirical pathway. The enrichment ratio of the compounds from the annotated peak tables was calculated with the average of the other empirical pathway size as the denominator.

### 4.6. Global Metabolic Network Visualization

The MS peaks from different studies were putatively annotated based on the Mummichog algorithm and mapped to the KEGG global metabolic network using the Peaks to Pathway module in MetaboAnalyst [59]. The sizes of the matched nodes (compounds) corresponded to the number of hits received from different studies. For those highlighted pathways, the corresponding compounds were extracted, with edges between compounds representing direct interactions based on the KEGG global metabolic reaction network.

### 4.7. Cluster Heatmap Analysis

The peak intensity tables from the individual datasets were uploaded to the Peaks to Pathway module in MetaboAnalyst 4.0 [59]. After normalization, the peak tables were displayed as an interactive heatmap with different clustering options. From the Overview on the left panel, we manually selected patterns of interest to be displayed on the Focus view on the central panel. Pathway activity predictions were performed based on Mummichog using the peaks in the current Focus view as significant peaks.

## 5. Conclusions

There are significant knowledge gaps in the systems biology of COVID-19. The ongoing multi-omics investigations will continue to yield valuable insights to fill this gap in the coming year. Global metabolomics can provide rich data that complement other omics layers to inform the development of diagnostics, prognostics, and treatment of COVID-19. In this study, we have systematically curated public metabolomics datasets and performed comprehensive data processing, analysis and meta-analysis to identify common as well as unique metabolic signatures underlying different clinical courses of COVID-19. Our results suggest that extensive dysregulations of amino acids metabolism, damage to the oxygen transport in red blood cells, exhaustion of endogenous immune bioactive metabolites and the suppression of multiple physiological processes are the metabolic characteristics underlying the progression of COVID-19. We will continue to improve the computational workflow and expand the scale and scope of the current meta-analysis when more metabolomics datasets become available in the coming year.

## Figures and Tables

**Table 1:** Summary of the seven datasets and the corresponding COVID-19 patient classifications.

Datasets	Chromatogram	MS	Patient Classification					Country
			Total	HC	MM	Severe	Fatal	
A1 [12]	UPLC-C18	Q/E	49	16	27	6	0	USA
A2 * [13]	UPLC-HILIC	Q/TOF	59	20	39	0	0	USA
A3 * [13]	UPLC-C18							
B1 [26]	HPLC- C18	micrOTOF	28	13	6	3	6	Brazil
C1 [8]	UPLC-C18	Triple TOF	76	26	37	11	2	China
C2 ** [9]	UPLC-C18	QE-HF	71	25	37	28	0	China
C3 ** [7]	UPLC- C30	Q/TRAP	96	10	14	11	9	China

HC: healthy control; MM: mild-to-moderate. * A2 and A3 are generated from the same samples using two different ultra-performance liquid chromatography (UPLC) columns. ** annotated peak tables.

## Data Availability

The data presented in this study are openly available from this link: https://drive.google.com/drive/folders/1R_I_gu5D3SkD_9q_J93HOA9GuKxZiGNG.

## Supplementary Materials

The following are available online at https://www.mdpi.com/2218-1989/11/1/44/s1. Table S1: Classification Standards for Different Severities of COVID-19; Table S2: Technical information of all datasets included in this study; Table S3: Clinical demographics characteristics of all subjects; Table S4: Optimized parameters of all datasets for raw spectral processing. Figure S1: The Spearman correlation analysis on the onset time (days) with the metabolites in the significantly perturbed pathways; Figure S2: Cluster heatmap analysis between COVID-19 and HC groups of Dataset A1; Figure S3: Cluster heatmap analysis between COVID-19 and HC groups of Dataset A2; Figure S4: Cluster heatmap analysis between COVID-19 and HC groups of Dataset A3; Figure S5: Cluster heatmap analysis between COVID-19 and HC groups of Dataset C1; Figure S6: Cluster heatmap analysis between COVID-19 and HC groups of Dataset C2; Figure S7: Cluster heatmap analysis between COVID-19 and HC groups of Dataset B1; Figure S8: Overview of perturbed pathways in COVID-19 across datasets for comparison between mild-to-moderate (MM) and severe COVID-19; Figure S9: The metabolic pattern between MM and severe of dataset C3; Figure S10: The metabolic pattern between MM and severe of dataset C1 and C2; Figure S11: Overview of perturbed pathways in COVID-19 across datasets for comparison between severe and fatal COVID-19; Figure S12: PRISMA 2009 Flow Diagram.
